# Takayasu Arteritis: A Difficult Diagnosis in a Patient With an Extensive Cardiovascular History

**DOI:** 10.7759/cureus.41256

**Published:** 2023-07-01

**Authors:** Carlos Peña, Niketa Kalara, Pallavi Velagapudi, Fernando Poli

**Affiliations:** 1 Internal Medicine, Mount Sinai Medical Center, Miami Beach, USA

**Keywords:** coronary artery bypass grafting(cabg), large vessel vasculitis and gca, takayasu disease, anginal chest pain, large vessel vasculitis

## Abstract

Large vessel vasculitides, such as Takayasu arteritis (TAK), are rare inflammatory conditions primarily affecting the aorta and its major branches. Its nonspecific symptoms and potential resemblance to atherosclerotic disease often pose diagnostic challenges. We present a case of a 57-year-old male with a history of extensive cardiovascular disease, initially attributed to atherosclerosis, resulting in several interventions, such as catheterization and major cardiac surgery, which didn't help improve his symptoms. Further evaluation revealed diffuse wall thickening of the aorta and its roots, as well as labs that suggested elevated inflammatory markers, comprehensive review of his chart and previous admissions, revealed that he had a well-documented aortitis for which he underwent a biopsy, which at the time was unrevealing. Furthermore, as he had significant aortic aneurysmal dilation, a thoracic cardiovascular surgeon remitted him to the rheumatology clinic, where he was placed on a prednisone taper and methotrexate regimen. Unfortunately, he redeveloped symptoms, and plans were made to transition to a tumor necrosis alpha (TNF-alpha) inhibitor. Our case highlights the importance of an accurate diagnosis and the prompt initiation of appropriate treatment in challenging cases of large vessel vasculitides. This case also underscores the need for heightened clinical awareness and interdisciplinary collaboration to ensure optimal patient care.

## Introduction

Primary large vessel vasculitis comprises two different conditions: Takayasu arteritis (TAK) and giant cell arteritis (GCA). Large vessel vasculitis manifests as inflammation of the aorta and its major branches, and non-specific symptoms often predominate [1]. The occurrence of large vessel vasculitides remains controversial, as there is a delay in diagnosis, making incidence studies unreliable; the annual incidence rate is estimated between 0.4 and 3.4 per million, with female predominance [2].

As per the Chapel Hill consensus, large vessels are defined as the aorta and its vessels except for the most distal branches. The two major entities affecting large vessels are giant cell arteritis and TAK [3]. GCA usually presents with cranial artery involvement and constitutional symptoms in patients above age 50, whereas TAK presents with vascular symptoms attributable to arteritis in addition to constitutional symptoms. TAK is also the leading cause of aortitis in young patients and complications such as heart failure, myocardial infarction, pulmonary hypertension, and ruptured aortic aneurysms are the major causes of morbidity and mortality in patients with Takayasu arteritis [4].

## Case presentation

A 57-year-old male presented to the emergency department (ED) 10 years ago with the chief complaint of retrosternal chest pain, described as sharp and radiating to the jaw and left upper extremity. Upon initial evaluation, he was noted to be hypertensive, around 180/115 mmHg, and his initial exam didn't reveal the presence of murmurs or diminished pulses; at the time, blood pressure was not checked on all limbs. The results from troponins prompted further workup due to a concern for non-ST-elevation myocardial infarction (NSTEMI). An echocardiogram showed normal wall motion and a preserved ejection fraction, however, since the patient’s symptoms persisted, he underwent a left cardiac catheterization, which revealed significant occlusion of the left anterior descending (LAD) artery, circumflex artery, and right coronary artery (RCA). Six drug-eluting stents were placed, and the patient was discharged home with dual antiplatelet therapy, lisinopril, metoprolol, and a statin.

Following this event, he experienced similar symptoms as in 2013, which prompted him to seek medical attention again. He underwent a left heart catheterization, which showed re-stenosis of the LAD and circumflex arteries in their distal portions. He required new stents to be placed; during this admission, an aneurysm was noted in his RCA, which was not present before.

Following his second admission, he presented to the ED several times in the setting of chest pain, which didn’t require admission since his symptoms responded well to nitroglycerin and his cardiac markers were negative. Two years after his first admission, he developed crushing retrosternal chest pain that awoke him in the middle of the night, radiated to the jaw and left arm, rated 10/10 in intensity, and associated with diaphoresis, which prompted another visit to the ED. He was admitted once again to the hospital; unfortunately, despite the administration of nitroglycerin, his symptoms didn’t improve. The cardiology service performed an angiography; this time, aneurysmal dilation of the thoracic aorta was seen, as re-stenosis of the previously treated vessels was once again noted. As a result of the aforementioned findings, he had to undergo a coronary artery bypass graft (CABG), and a biopsy from the dilated aorta was taken.

The pathology revealed a portion of mostly adventitial vascular tissue containing focally dense infiltrates of predominantly lymphocytes and fewer plasma cells and histiocytes, mostly adventitial/periaortic. Focally, however, some chronic inflammatory cells and histiocytes were noted in the aortic media, although it was confusing for the pathologist, as there was underlying fibrosis of the tissue; nevertheless, no giant cells, granulomas, or cystic medial necrosis were noted. The final report from the biopsy was read as unspecific chronic inflammation, although there was a comment made at the time that the presence of vasculitis couldn't be excluded.

**Figure 1:**
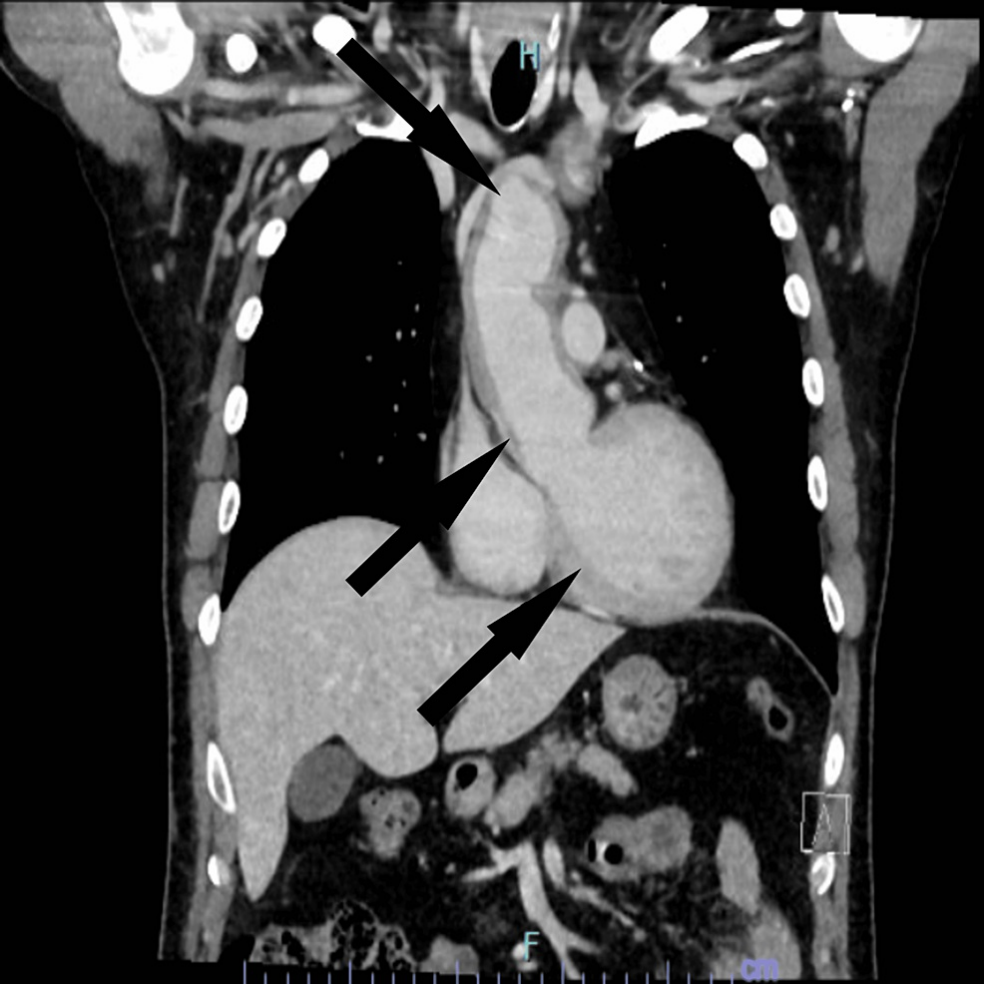
Computed tomography angiography of the chest showing thickening of the walls of the aorta and aneurysmal dilation

In the following years, the patient persisted with symptoms despite having proper medical therapy, a routine echocardiogram revealed worsening of his previously dilated ascending aorta and root; as a result, he was referred to cardiothoracic surgery for further evaluation. Upon evaluating the patient, a computed tomography angiography (CTA) of the chest was ordered, which revealed diffuse wall thickening throughout the thoracic aorta, and to a lesser extent, the proximal arch vessels with circumferential wall thickening most prominent at the proximal left subclavian artery and aneurysmal dilation of the aortic root, ascending aorta, and transverse and descending aorta (Figure 1). Incidentally imaged abdominal aorta during this study also exhibited circumferential wall thickening of the vessel.

Additionally, the thickening evidenced across the aorta was also observed in his coronary vasculature. The question of large vessel vasculitis was posed once again since some of the features were concerning for aortitis due to an underlying inflammatory process. Rheumatology was consulted for suspicion of Takayasu arteritis.

On initial evaluation, the patient admitted experiencing episodic chest pain, which partially responded to nitroglycerin. He denied arthralgias, myalgias, weight loss, or fevers; however, he admitted to experiencing persistent fatigue. On physical exam, there were prominent S1 and S2 on auscultation, a soft 1/6 systolic murmur auscultated over the left parasternal border, his pulses were present on all limbs. Labs were done on initial evaluation (Table 1).

**Table 1:** Labs on the first visit to the clinic

Test	Result	Reference Range
Protein, Total	8.6 g/dL	6.1-8.1 g/dL
Albumin	3.9 g/dL	3.8-4.8 g/dL
Alpha 1 Globulin	0.4 g/dL	0.2-0.3 g/dL
Alpha 2 Globulin	0.9 g/dL	0.5-0.9 g/dL
Beta 1 Globulin	0.5 g/dL	0.4-0.6 g/dL
Beta 2 Globulin	0.8 g/dL	0.2-0.5 g/dL
Gamma Globulin	2.2 g/dL	0.8-1.7 g/dL
ANCA Screen	Negative	Negative
Myeloperoxidase Antibody	<1.0 AI	<1.0 AI
Proteinase-3 Antibody	<1.0 AI	<1.0 AI
Immunoglobulin A	708 mg/dL	47-310 mg/dL
Immunoglobulin G	2345 mg/dL	600-1640 mg/dL
Immunoglobulin M	95 mg/dL	50-300 mg/dL
Immunoglobulin G Subclass 4	309.6 mg/dL	4.0-86.0 mg/dL
Sedimentation Rate by Modified Westergren	58 mm/h	< OR = 20 mm/h
C-Reactive Protein	33.4 mg/L	<8.0 mg/L
White Blood Cell Count	8.6 Thousand/uL	3.8-10.8 Thousand/uL
Red Blood Cell Count	5.17 Million/uL	4.20-5.80 Million/uL
Hemoglobin	15.8 g/dL	13.2-17.1 g/dL
Hematocrit	46.4 %	38.5-50.0 %
Mean Corpuscular Volume	89.7 fL	80.0-100.0 fL
Mean Corpuscular Hemoglobin	30.6 pg	27.0-33.0 pg
Mean Corpuscular Hemoglobin Concentration	34.1 g/dL	32.0-36.0 g/dL
Red Cell Distribution Width	14.2 %	11.0-15.0 %
Platelet Count	338 Thousand/uL	140-400 Thousand/uL
Mean Platelet Volume	9.8 fL	7.5-12.5 fL
Absolute Neutrophils	4893 cells/uL	1500-7800 cells/uL
Absolute Lymphocytes	2632 cells/uL	850-3900 cells/uL
Absolute Monocytes	722 cells/uL	200-950 cells/uL
Absolute Eosinophils	292 cells/uL	15-500 cells/uL
Absolute Basophils	60 cells/uL	0-200 cells/uL

His initial labs suggested the presence of an autoimmune disorder, as it was impressive for inflammation because both the erythrocyte sedimentation rate and C-reactive protein were high, in conjunction with increased immunoglobulins. This once again supported part of the thought process, suggesting that Takayasu arteritis was the reason why he had experienced several cardiovascular complications for the last 10 years of his life.

**Figure 2:**
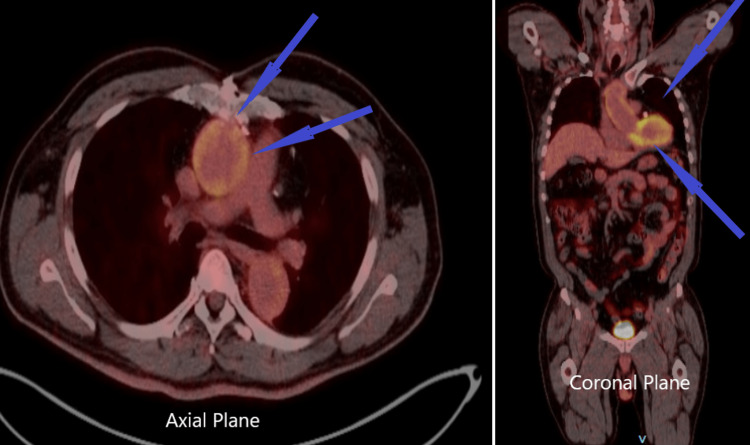
Axial planes and coronal planes of PET computed tomography, revealing moderately increased uptake of the aorta and its vessels PET: positron emission tomography

The patient was started on prednisone 60 mg, methotrexate 15 mg, and folic acid. His inflammatory biomarkers continued to be monitored since cardiothoracic surgery had plans for repairing the aneurysm once inflammation in the vessels improved. As part of the workup done, a positron emission tomography (PET) confirmed the presence of active inflammation (Figure 2).

Despite showing clinical improvement in the following weeks, he developed symptoms again, which prompted a new visit to the ED. Following this event, he was re-evaluated at the clinic and was placed again in a new steroid taper to better control his symptoms. As there was a concern for a high recurrence of the disease, he was started on adalimumab to help achieve remission, as his response to methotrexate in conjunction with steroids was not the one expected and his presentation was highly suspicious of refractory TAK.

## Discussion

There are no universal criteria for diagnosing large vessel vasculitides, including GCA and TAK. A timely diagnosis can be established by considering the physical exam, laboratory panels, and imaging [5]. Takayasu arteritis typically presents in three phases. The first one consists of nonspecific symptoms, such as fever and fatigue, and in the second one, we can appreciate vascular mural inflammation, which results might result in carotidynia as a result of carotid involvement or dorsal pain if the thoracic aorta is inflamed. The third phase is characterized by narrowing or occlusion in the proximal part of the arterial branches originating from the aortic arch; as a result, absent pulses can be found during the physical exam, as well as intermittent limb claudication [6].

Coronary vasculitis, although extremely rare, should be considered when seen in young patients with unexplained acute coronary syndrome (ACS), congestive heart failure (CHF), or patients with a known history of vasculitis. Typically, it presents as stenosis, occlusion, or aneurysmal dilation or rupture of the coronaries [7]. In the case of our patient, by the time he was evaluated at the rheumatology clinic, he had developed several cardiac complications resulting from chronic inflammation that was thought to be solely attributed to atherosclerotic disease. Several biomarkers have been used to aid the diagnosis such as cytokines, including interleukin-6 (Il-6) and TNF-alpha, and acute phase reactants, such as erythrocyte sedimentation rate (ESR) and C-reactive protein (CRP); however, fewer markers that accurately reflect vascular damage have been described. Recently, the enhanced liver fibrosis (ELF) score has been described as a promising alternative to assess vascular damage [8]. The ELF score consists of the evaluation of procollagen-III aminoterminal propeptide (PIIINP), tissue inhibitor of matrix metalloproteinase-1 (TIMP-1), and hyaluronic acid (HA). Liver fibrosis is a dynamic state that reflects an imbalance between metalloproteinases and tissue inhibitors of metalloproteinases, resulting in the deposition of collagen fibers. As one of the main hallmarks of Takayasu arteritis is endothelial damage, it has been hypothesized that this scoring system could accurately quantify vascular damage [9].

Recent findings seem to suggest that imaging has largely replaced histology to diagnose large vessel vasculitides. Among the studies that have been used for diagnosing TAK, we can mention ultrasound (US), computed tomography angiography (CTA), magnetic resonance angiography (MRA), and 18F-fluorodeoxyglucose positron emission tomography (18-FDG-PET). Ultrasound has proved to be a convenient diagnostic tool, as it is inexpensive and can be done during the physical exam. As this disease courses with significant vessel inflammation, thickening of the affected vessels are expected to be seen, which will also be the case with CTA and MRA, with these two providing a better characterization of occlusion and ectasia of the vessels. Lastly, the PET scan will provide evidence of increased glucose uptake in inflamed arteries, providing a better characterization of the vessels affected and outlining the disease extent. However, one significant limitation to keep in mind is the significant cost of this study [10]. For our patient in question, the CTA revealed aneurysmal dilation of the aorta and significant wall thickening, ultimately the PET scan confirmed increased uptake on the affected vessels confirming the diagnosis of TAK.

As our patient had a biopsy that at the time it was done, it didn't entirely exclude the diagnosis of vasculitis, it is important to mention that presently, the biopsy is particularly difficult to perform, as most of the arteries affected are extracranial. In this case, it is advisable to harvest the material in the event of surgery, which was the case when he had a CABG [9]. One of the findings most commonly associated with large vessel vasculitides is aortitis; however, there are other autoimmune causes, such as Behcet's disease, ankylosing spondylitis, rheumatoid arthritis, lupus, and immunoglobulin G4 (IgG4) related disease that could potentially account for these findings. For example, in the case of Behcet's disease, the patients may also present with recurrent oral and genital ulcers and uveitis, whereas in ankylosis spondylitis, back pain is a predominant feature as well as the constitutional symptoms, which is also the case in lupus, where many of the symptoms involve several organs and systems. Furthermore, IgG4-related disease courses with pancreatitis, salivary gland enlargement, and retroperitoneal fibrosis [11].

Aortitis can also be the result of infections. Generally, it occurs in elderly patients with pre-existing atherosclerotic disease, aneurysmal disease, or infective endocarditis. Among the most common agents involved, it is important to mention *Treponema pallidum*, *Staphylococcus* species, *Enterococcus* species, *Salmonella,* and exceptionally tuberculosis [12]. As it was explained, there are many causes accounting for aortitis, hence, a thorough history and physical exam are required when approaching this type of patient.

In an effort to facilitate a timely diagnosis, the American College of Rheumatology (ACR) and the European Alliance of Associations for Rheumatology (EULAR) provided a classification for Takayasu arteritis in 2022 in an attempt to better distinguish it from other entities. Among the absolute requirements for this classification, the patient must be below age 60 and the imaging findings must have signs of vasculitis. Furthermore, other items consisted of a scoring system that takes into account findings in the physical exam as well as additional imaging criteria related to the involvement of vascular territories. Although promising, this new system is recommended once alternative diagnoses have been excluded and if there is already an established diagnosis of medium or large vessel vasculitis [13]. In the case of our patient, he was diagnosed at age 57, had imaging findings suggestive of vasculitis, and had involvement of three vascular territories, which comprised the ascending aorta, the left subclavian arteries, and although partially imaged, there was wall thickening of the abdominal aorta, which sums a total of 5 points, fitting into the classification of Takayasu arteritis as proposed by the ACR/EULAR.

Treatment will initially consist of a high-dose glucocorticoid (40 mg to 60 mg daily prednisone equivalent) for induction of remission, with a goal of tapering to a dose of 10 mg to 20 mg daily in the upcoming three months and ultimately to less than 10 mg/day in a year. However, the taper should take into consideration the risk of relapsing, which will result in having to re-increase the dosing should this happen. Additionally, non-biologic disease-modifying agents (DMARDs) should be given in conjunction with glucocorticoids. Several agents have been used to achieve remission, such as methotrexate, leflunomide, mycophenolate mofetil, and cyclophosphamide. The use of biologic DMARDs, such as tocilizumab and TNF-alpha inhibitors, is typically reserved for patients experiencing relapses [14,15]. The ACR recommends conventional oral glucocorticoid therapy over IV glucocorticoid pulse therapy, as there is no clear evidence of superior efficacy. In addition, there is the encouragement of using conventional steroid-sparing medication over tocilizumab, and this last one is not recommended over TNF-alpha inhibitors; it is only reserved for patients failing remission [16]. In the case of our patient, he was started on methotrexate and glucocorticoids but his response was not the one expected, as he redeveloped symptoms shortly after. The decision to transition him to adalimumab was taken in addition to increasing the dosing in his steroid taper, as this is the management that is widely proposed as per guidelines.

Ultimately, the role of antiplatelet therapy should only be reserved for patients with a high risk for ischemic events or active cardiovascular disease. Especially in the setting of flow-limiting vertebrobasilar disease, its use should be considered on an individual basis, as it will increase the risk of bleeding [14,16].

## Conclusions

Takayasu arteritis poses a challenging diagnostic dilemma, lacking specific biomarkers. However, the recent classification provided by the European League Against Rheumatism has expanded the age group in which this rare disease occurs. This expanded range aids in considering Takayasu arteritis as a potential differential diagnosis. Notably, its symptoms can resemble those of atherosclerotic cardiovascular disease, leading to potential oversight during patient evaluation. Nevertheless, persistent consultations for recurrent chest pain, despite adherence to prescribed medications for coronary artery disease, should serve as a red flag, prompting consideration of this rare vasculitis.

During the discussion, it was emphasized that various imaging modalities can aid in establishing a diagnosis. However, limitations associated with cost may arise. Therefore, it is prudent to consider the routine use of ultrasound as a cost-effective tool that may help save time and resources before resorting to more expensive studies. Given the propensity for relapse in this disease, close patient monitoring is crucial to prevent the development of life-threatening complications. Additionally, as mentioned earlier, biopsies play a valuable role in providing diagnostic insights although their feasibility may be limited. Thus, a meticulous approach to history taking and physical examination becomes essential for ensuring a timely diagnosis and the initiation of appropriate therapy at the earliest opportunity.
